# In Vivo and In Vitro Hepatoprotective Effects of* Geranium koreanum* Methanolic Extract via Downregulation of MAPK/Caspase-3 Pathway

**DOI:** 10.1155/2017/8137627

**Published:** 2017-07-05

**Authors:** Md Rashedunnabi Akanda, In-Shik Kim, Dongchoon Ahn, Hyun-Jin Tae, Weishun Tian, Hyeon-Hwa Nam, Byung-Kil Choo, Byung-Yong Park

**Affiliations:** ^1^College of Veterinary Medicine and Biosafety Research Institute, Chonbuk National University, Iksan 54596, Republic of Korea; ^2^Department of Pharmacology and Toxicology, Sylhet Agricultural University, Sylhet 3100, Bangladesh; ^3^Department of Crop Science & Biotechnology, Chonbuk National University, Jeonju 54896, Republic of Korea

## Abstract

*Geranium koreanum* (GK) is an indigenous Chinese herbal medicine widely used for the treatment of various inflammation and liver disorders. However, the exact mechanism of action of GK remains unknown. This study aimed to investigate the protective effect and related molecular mechanism of GK on NaAsO_2_-induced cytotoxicity in HepG2 cells and liver damage in mice. The cytoprotective role of GK was assessed on HepG2 cells using MTT assay. Oxidative stress and lactate dehydrogenase levels were measured with ROS and LDH assay. Histopathology and serum enzymes levels were estimated. The molecular mechanism was evaluated by qPCR and immunoblotting to ensure the hepatoprotective role of GK against NaAsO_2_ intoxication in mice. We found cotreatment with GK significantly attenuated NaAsO_2_-induced cell viability loss, intracellular ROS, and LDH release. Hepatic histopathology and serum biochemical parameters, ALT, and AST were notably improved by cotreatment with GK. Beside, GK markedly altered both mRNA and protein expression level of MAPK. The proapoptotic and antiapoptotic protein Bax/Bcl-2 ratio was significantly regulated by GK. Moreover, GK remarkably suppressed the postapoptotic transcription protein cleaved caspase-3 expression. The present study reveals that GK possesses hepatoprotective activity which is probably involved in the modulation of the MAPK/caspase-3 pathway.

## 1. Introduction

Arsenic is a naturally occurring metalloid toxicant that is known to contaminate groundwater due to its geographical structure. Environmental exposure to the inorganic arsenic compound is a global health concern for human and animal, especially in countries such as Bangladesh, India, and Pakistan [1, 2]. Drinking water and industrial effluence are the leading sources of human exposure to inorganic arsenic [3]. The inorganic form of arsenic is more harmful than the organic state, and the most toxicologically effective arsenic compounds are in the trivalent oxidation state, mainly arsenic trioxide (As_2_O_3_) and sodium arsenite (NaAsO_2_) [4, 5]. Continued exposure to inorganic arsenic is associated with increased risk of liver, lung, skin, and kidney cancer. The liver is a major site of arsenic localization and biotransformation and, therefore, is affected by high arsenic level. Oxidative stress, genotoxicity, alterations in DNA methylation, cell proliferation, and induction of cell death have been reported followed by arsenic exposure [6, 7]. This stress leads to damage of essential biomolecules and organs and may have a significant impact on the whole organism; the triggering of liver diseases serves as key pathophysiological evidence of this damage [8]. Oxidative stress is a widely standard hypothesis for NaAsO_2_-induced cytotoxicity [9]. Notably, generation of intracellular ROS level depends on the NaAsO_2_ exposure concentration and its biological effects on cells. Exposure to a lower concentration of NaAsO_2_ (0.5–5 *μ*M) induces excess oxidative stress in cells [6], while exposure to relatively higher concentration of NaAsO_2_ (20–100 *μ*M) causes apoptotic cell death [10].

ROS are highly reactive molecules which can damage a different cellular structure such as DNA, proteins, and lipids and alter their functions [11]. A proper balance between the generation of intracellular ROS and antioxidants is known to be vital for regulation of the biological consequences. Arsenic-mediated oxidative stress leads to adverse biological processes, including cell death following stimulation of MAPK and apoptotic signals [12, 13]. Sulfhydryl-reactive compound NaAsO_2_ activates MAPK pathway and concurrently nuclear translocation of transcription factors which modify gene expression through phosphorylation-dependent substrate stimulation [13, 14]. MAPK cascade consists of three major kinases: ERK, JNK, and p38. Particularly, the activation of JNK and p38 activates apoptosis in response to oxidative stress and DNA damage [15]. Also, ERK activity promotes apoptosis by induction of mitochondrial cytochrome *c* release [16]. Beside, Bcl-2 family members provide the most important controlling mechanism for apoptosis and a major site of their action is the mitochondria. The relationship between the proapoptotic protein, Bax, and the antiapoptotic protein, Bcl-2, regulates mitochondria-dependent apoptotic cell death by activating the downstream transcription protein caspase-3 [17]. Caspase activation is under the direct control of kinases and the indirect control of phosphorylation via regulation of apoptotic protein [18]. Hence, DNA fragmentation and protein phosphorylation lead to triggering of caspase and serine/threonine protein phosphorylation activation [19]. Thus, the inhibition of apoptotic signaling pathway activated by NaAsO_2_-induced oxidative damage may be an excellent strategy for treatment of hepatic disorders.

Phytomedicine and active ingredients from plants have received considerable attention; these antioxidant agents may offer some protection against oxidative stress and play a potential role in reducing the toxicity of trivalent arsenite [20]. Traditional herbal medicine is a potential source for effective and alternative remedies for liver diseases [21]. Antioxidants are able to donate electrons and neutralize free radicals resulting in the prevention of cell injuries [22]; however, there is limited information on the molecular mechanism and biological activities of these compounds. Recently, the search for effective and natural phytocompounds with antioxidant activity has been strengthened [23]. We describe here for the first time a simple and reliable process for obtaining an extract of GK with which to synthesize a solution with a strong effectiveness against NaAsO_2_-induced liver toxicity both in vivo and in vitro. Traditionally GK extract has been used as Chinese traditional medicine to treat many diseases such as itching, bruising, enteritis, chronic diarrhea, and liver disorder and GK contains widely tannins, quercetin, gallic acid, and succinic acid [24]. An earlier study reported that GK extract shows antibacterial activities [24]. Since antioxidant properties are important factors in the management of liver disease, thus GK is suggested to be an effective hepatoprotective medicine. Therefore, based on the traditional uses and pharmacological activities of GK, our study investigates the hepatoprotective role of GK on NaAsO_2_-induced oxidative damage in vitro and in vivo.

## 2. Materials and Methods

### 2.1. Chemicals and Antibodies

Sodium arsenite (NaAsO_2_), 3-(4,5-dimethylthiazol-2-yl)-2,5-diphenyltetrazolium bromide (MTT), gallic acid, rutin, penicillin/streptomycin, hematoxylin, eosin, and protease inhibitor were purchased from Sigma-Aldrich (St. Louis, MO, USA). Fetal bovine serum (FBS), Dulbecco's modified Eagle's medium (DMEM), and other cell culture reagents were obtained from Gibco (Carlsbad, CA, USA). Dimethyl sulfoxide (DMSO) was from Bioshop (Canada). RNA extraction kits were purchased from RiboEx and Hybrid-R (Gene All, South Korea). Tissue protein extraction (T-PER), cDNA synthesis (ReverTra Ace qPCR RT Kit), and BCA protein assay kits were purchased from Thermo Scientific (Waltham, Massachusetts, USA). SYBR green qPCR kit was purchased from TOYOBO (Japan). Primary antibodies (phospho-ERK1/2, phospho-JNK phospho p38, Bax, Bcl-2, cleaved caspase-3, and caspase-3) and *β*-actin were purchased from Cell Signaling, Danvers, MA, USA. Secondary antibody (goat anti-rabbit IgG-HRP) was purchased from Santa Cruz (Santa Cruz, CA, USA). ECL detection kit was acquired from Abfrontire (South Korea), and the ALT and AST kits were from ASAN (South Korea). Lactate dehydrogenase (LDH) cytotoxicity assay kit was obtained from TAKARA (Japan), and the ROS detection kit was from Promega (USA). Zoletil 50 was supplied by Virbac S.A. (France).

### 2.2. Plant Materials and Extraction

The whole plant of GK was collected from the Crop Science and Biotechnology Laboratory, College of Agriculture and Life Science, Chonbuk National University, South Korea. The dried plant was boiled 3 times in 100% methanol for 2 h. The extract was filtered, concentrated under vacuum, and dried with a lyophilizer. The powdered extract was dissolved in DMSO and sterilized by filtering through a 0.22 *μ*m syringe filter. The maximum concentration of DMSO used for in vitro studies was 0.1%. For in vivo study, GK extract was dissolved in distilled water. The dried extract was kept in −20°C.

### 2.3. Determination of Total Phenolic and Flavonoid Content

Total phenolic and flavonoid content of GK extract was measured according to the previously described method [25].

### 2.4. Cell Culture

HepG2 cells were maintained at 37°C in a 5% CO_2_ humidified incubator. Cells were cultured in DMEM enriched with 10% FBS and 1% penicillin and streptomycin. The culture media were changed every 2 days and subcultured when cells reached about 90% confluency.

### 2.5. Evaluation of Cell Viability

The MTT assay provides a sensitive determination of the metabolic status of cellular mitochondrial enzymes. We did MTT assay according to a previously described method with slight modification [26]. HepG2 cells (1 × 10^4^ cells/well in 96 well plates) were cultured at 37°C for 24 h with 10% FBS prior to experimental treatment. For evaluating the cytotoxicity of GK extract, cells were treated with GK (1, 5, 10, 20, and 40 *μ*g/mL) for 24 h. In contrast, measuring the cell viability, cells were pretreated for 1 h with different concentration of GK (5, 10, and 20 *μ*g/mL) in FBS-free media and then cotreated with GK and NaAsO_2_ (10 *μ*M) for an additional 24 h. The culture medium was replaced by 0.5 mg/mL MTT solution and cultured for another 2 h. MTT solution was carefully aspirated and blue formazan solubilized in DMSO. OD was measured at a wavelength of 570 nm using a tunable* versa max *microplate reader (Molecular Device, USA).

### 2.6. Measurement of Intracellular ROS and LDH

Intracellular ROS generation and LDH release into the culture medium by dead cells were determined using the ROS detection kit and LDH cytotoxicity assay kit, according to manufacturer instructions. HepG2 cells (1 × 10^4^ cells/well in 96 well plates) were cultured at 37°C for 24 h. After adherence, cells were pretreated with GK (5, 10, and 20 *μ*g/mL) for 1 h and followed by cotreatment with 10 *μ*M NaAsO_2_ and GK for another 24 h. Absorbance was measured at 490 nm using a tunable* versa max* microplate reader.

### 2.7. Animal Management and Experimental Design

6-week-old ICR mice were maintained in accordance with the animal welfare regulation of the Institutional Animal Care and Use Committees, Chonbuk National University Laboratory Animal Centre, South Korea. Mice were kept in standard mouse cages with an ad libitum supply of food and distilled water. Temperature (23 ± 2°C), humidity (35–60%), and photoperiod cycle (12 h light and 12 h dark) were maintained over the experimental period. Mice were adapted to the laboratory atmosphere for 1 week before starting the experiment. We followed previously established method for in vivo study [27]. Briefly, a total 48 mice were randomly divided into four groups: (1) normal control mice were treated with normal saline, (2) toxic control mice were treated with NaAsO_2_ once daily (10 mg/kg body weight, p.o., for 10 days), (3) experimental mice were treated with GK extract once daily (20 mg/kg body weight, p.o., for 15 days) prior to treatment with NaAsO_2_ (10 mg/kg body weight, p.o., for 10 days), and (4) experimental mice were treated with GK extract once daily (20 mg/kg body weight, p.o., for 15 days). After the experimental period, mice were fasted overnight and sacrificed under anesthetized using Zoletil 50. Blood samples were collected for serum biochemical analyses. Liver samples were collected and immediately fixed with 10% NBF for histopathological analysis and kept in −80°C for qPCR and immunoblotting analyses.

### 2.8. Histopathological Analysis of Liver

Liver samples were excised for histopathological evaluation. Pieces of the same lobe of liver from each mouse were immediately fixed in 10% NBF. Samples were processed in an autoprocessor (Excelsior ES, Thermo Scientific, USA). After embedding in paraffin, 5 *μ*m sections of liver tissue were stained with hematoxylin and eosin and mounted on a glass slide. Staining by hematoxylin and eosin was utilized to explore histological architecture and apoptotic changes among hepatocytes. Digital images were obtained using a Leica DM2500 microscope (Leica Microsystems, Germany) at a fixed 200x magnification. The diameter of the portal vein was measured using image measurement software (iSolution DTM).

### 2.9. Serum Biochemical Assessment

For assessment of liver function, sera were collected from experimental mice. We followed previously recognized technique for serum separation [28]. At first, blood samples were collected directly from the heart and incubated for 30 min at room temperature. Whole blood was centrifuged at 3000 rpm for 15 min at 4°C to collect the sera, after which serum biochemical components including ALT and AST were analyzed according to the manufacturer reference.

### 2.10. qPCR Analysis

Total RNA was extracted from liver tissue according to the manufacturer's instructions, and RNA concentration was quantified using a BioSpec-nanospectrophotometer (Shimadzu Biotech, Tokyo, Japan) at a 260/280 nm ratio. All RNA samples were stored at −80°C until use. For cDNA synthesis, total RNA (3 *μ*g) was used, and the cDNA synthesis procedure was performed according to the manufacturer's instructions. qPCR was performed using the SYBR Green Real-Time PCR master mix with the Roche LightCycler™, and the following conditions were maintained to complete 40 cycles: predenaturation 95°C for 30 sec, denaturation 95°C for 5 sec, annealing 55°C for 10 sec, and extension for 72°C for 15 sec. The qPCR reaction system was performed according to manufacturer's instructions. The relative mRNA expression was analyzed by normalized the target mRNA value and *β*-actin. The nucleotide sequences of the primers are presented in Table 1 [29].

### 2.11. Immunoblotting Analysis

For immunoblotting analysis, liver tissue lysates were prepared in lysis buffer: containing T-PER, PMSF, Na3VO4, and protease inhibitor cocktail. The total protein concentration of lysed liver was measured using the BCA protein assay kit. An equal amount of protein was separated by 12% SDS-PAGE and transferred to a nitrocellulose membrane. The membrane was blocked with 5% BSA or 5% nonfat skim milk in TBST or PBST for 2 h at room temperature followed by incubation with primary antibodies against pERK, pJNK, pp38, Bax, Bcl-2, caspase-3, cleaved caspase-3, and *β*-actin overnight at 4°C. The blot was washed and then incubated with secondary antibody for 2 h. Proteins were detected using an ECL detection kit, and images were obtained using a UV imaging system (LAS-400 image system, GE Healthcare, UK). *β*-Actin was used as the control.

### 2.12. Statistical Analysis

Data were analyzed with Graph Pad Prism 5.0 and expressed as mean ± SEM. Group comparisons were performed using one-way and two-way analysis of variance (ANOVA) followed by Bonferroni post hoc tests. The minimum statistical significance was considered at *p* < 0.05 for all analyses.

## 3. Results

### 3.1. Analysis of Total Phenolic and Flavonoid Content of GK

Phenolic and flavonoid contents are the secondary metabolites of plant which exhibits a series of biological activities and certainly, possessing antioxidant properties. The total phenolic and flavonoid contents of GK extract were investigated and presented in Table 2.

### 3.2. GK Reduced NaAsO_2_-Induced Cytotoxicity in HepG2 Cells

**Figure 1:**
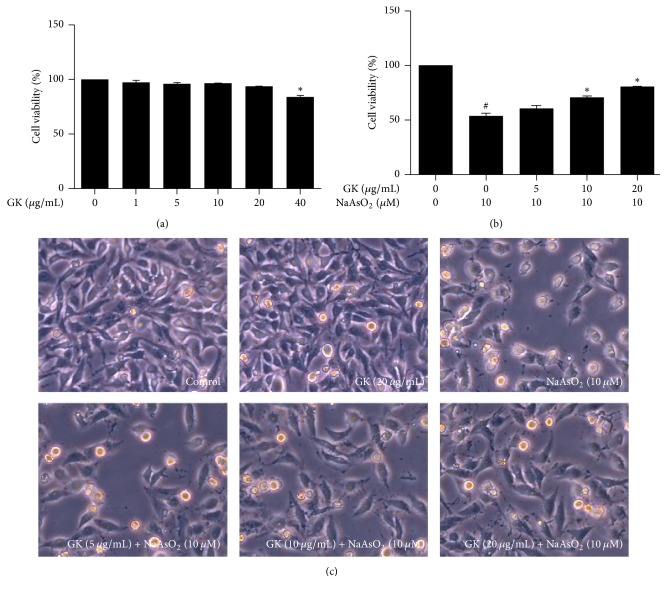
Hepatoprotective effects of GK against NaAsO_2_-induced toxicity in HepG2 cells. (a) Cytotoxicity and (b) cell viability were determined by MTT assay and (c) observation of HepG2 cell morphology. Cells were pretreated with different concentration of GK for 1 h, followed by cotreatment with 10 *μ*M NaAsO_2_ for 24 h. ^#^*p* < 0.05 when compared with the control and ^*∗*^*p* < 0.05 when compared with NaAsO_2_ treated. Data are expressed as mean ± SEM from triplicate experiments.

We performed MTT assay to evaluate the mechanism responsible for the hepatoprotective effects of GK against NaAsO_2_-induced damage in HepG2 cells. To investigate the cytotoxicity of GK extract, the MTT assay was done using a different concentration of GK extract (1, 5, 10, 20, and 40 *μ*g/mL) for 24 h. Our data showed that cell viability was not significantly altered by incubation with the GK extract (1, 5, 10, and 20 *μ*g/mL) for 24 h while GK 40 *μ*g/mL significantly decreased the cell viability (Figure 1(a)). For cell viability, at the end of the 24 h treatment period, incubation with 10 *μ*M NaAsO_2_ alone reduced cell viability (%) to 53.55 ± 2.797 compared to control. This cytotoxic effect was attenuated by cotreatment with GK in a dose-dependent manner; cotreatment with GK (5, 10, and 20 *μ*g/mL) enhanced cell viability (%) 60.65 ± 2.873, 70.52 ± 1.439, and 80.45 ± 2.088, respectively (Figure 1(b)). Similarly, observations of cells morphology with light microscopy demonstrated that a number of cells became pyknotic and apoptotic with NaAsO_2_ alone compared to GK cotreated groups. The pyknotic and apoptotic cell populations were reduced in the GK extract cotreated groups in a dose-dependent manner (Figure 1(c)). Together, these results indicate that GK had hepatoprotective effects against NaAsO_2_-induced cytotoxicity in HepG2 cells.

### 3.3. GK Inhibited ROS and LDH Release in HepG2 Cells

**Figure 2:**
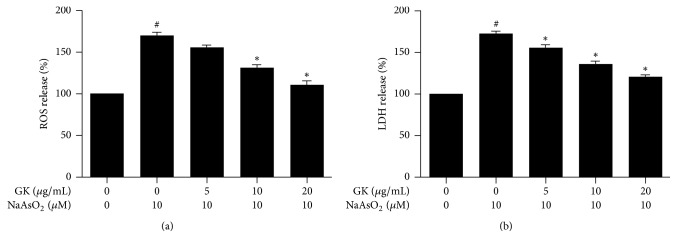
GK extract suppressed the release of intracellular ROS and LDH in HepG2 cells. (a) ROS and (b) LDH levels were measured. Cells were pretreated with different concentration of GK for 1 h, followed by cotreatment with 10 *μ*M NaAsO_2_ for 24 h. ^#^*p* < 0.05 when compared with the control and ^*∗*^*p* < 0.05 when compared with NaAsO_2_ treated. Data are expressed as mean ± SEM from triplicate experiments.

Experimental evidence indicates that exposure to arsenic induces oxidative stress [30]. The hepatoprotective effect of GK was confirmed with ROS and LDH assays. After treatment with 10 *μ*M NaAsO_2_ for 24 h, there was a significant increase in intracellular ROS generation (%) to 169.65 ± 4.268 of control and LDH release (%) up to 172.77 ± 2.846 of control. However, cotreatment with GK notably reduced intracellular ROS and LDH level. At GK concentrations of 5, 10, and 20 *μ*g/mL, intracellular ROS (%) decreased to 155.06 ± 3.193, 131.36 ± 3.364, and 110.44 ± 4.908, respectively (Figure 2(a)), and LDH release (%) significantly declined to 154.89 ± 3.345, 135.63 ± 3.367, and 121.03 ± 2.002, respectively (Figure 2(b)). Together, these data indicate that GK protected HepG2 cells from the damaging oxidative effects caused by NaAsO_2_.

### 3.4. GK Improved the Histopathology of Liver and Body Weight

**Figure 3:**
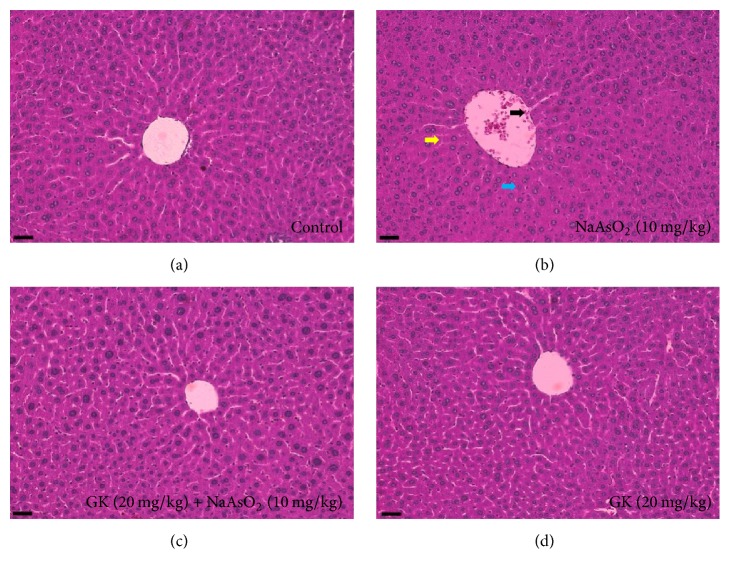
GK improved liver histology in experimental mice. Untreated mice were kept as a control to compare histological changes induced by NaAsO_2_. (b) NaAsO_2_ (10 mg/kg) treated mice liver. The black arrow indicates the dilated portal vein, the pale green arrow indicates the degenerative nucleus, and the yellow arrow indicates blurred cytoplasm. In contrast, cotreatment with GK (20 mg/kg) improved histological changes as compared to NaAsO_2_ alone. Scale bar 200 *μ*M.

**Figure 4:**
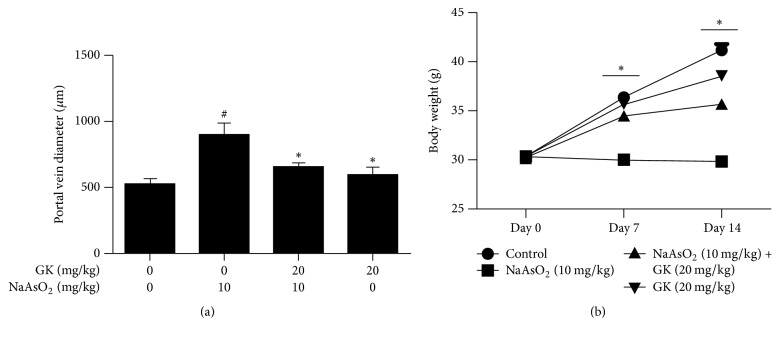
GK reduced the portal vein diameter and increased the body weight in experimental mice. (a) The diameter of the portal vein was increased in NaAsO_2_ (10 mg/kg) treated liver. Cotreatment with GK (20 mg/kg) significantly decreased the portal vein diameter and (b) the average body weight of the GK cotreated group was significantly increased as compared to the NaAsO_2_ alone in day 7 and day 14. ^#^*p* < 0.05 when compared with the control and ^*∗*^*p* < 0.05 when compared with NaAsO_2_ treated. Data are expressed as mean ± SEM.

Microscopic analysis of the liver revealed normal hepatocyte structures arrange around the portal vein in both control and GK cotreated group. Examination of mice liver from the NaAsO_2_ treated group (10 mg/kg) showed slight hydropic and degenerative nuclei, cellular infiltration, and dilation of the portal vein. Histopathological changes induced by NaAsO_2_ were remarkably improved by cotreatment with GK (20 mg/kg), and in addition, GK (20 mg/kg) alone did not show damaging effects in liver tissue (Figure 3). The portal vein diameter significantly reduced by the GK cotreatment (Figure 4(a)). Besides, the body weight of mice was significantly increased at day 7 and day 14 in the GK cotreated group compared to the NaAsO_2_ alone (Figure 4(b)).

### 3.5. GK Treatment Restored Serum Liver Enzymes Activity

**Figure 5:**
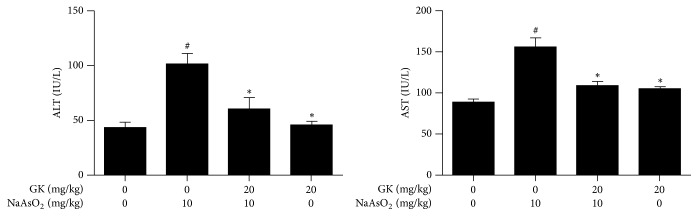
Effects of GK on serum biochemical parameters in experimental mice. Both ALT and AST were increased in NaAsO_2_ (10 mg/kg) intoxicated mice. Cotreatment with GK (20 mg/kg) significantly decreased the serum ALT and AST level. ^#^*p* < 0.05 when compared with the control and ^*∗*^*p* < 0.05 when compared with NaAsO_2_ treated. Data are expressed as mean ± SEM.

Serum biochemical parameters serve as an important indicator of physiological abnormalities in liver intoxication. We found that the levels of the serum cytosolic enzymes ALT (101.58 ± 9.473 IU/L) and AST (155.75 ± 11.461 IU/L) were considerably higher in NaAsO_2_-intoxicated mice than in normal controls (ALT, 43.39 ± 4.916 IU/L, and AST, 88.83 ± 4.375 IU/L). Cotreatment with GK significantly improved liver function by reducing ALT and AST level (60.29 ± 10.160 IU/L and 108.40 ± 5.061 IU/L, resp.) as compared to NaAsO_2_ alone (Figure 5). These data indicate that GK protected against the hepatic damaging effects caused by NaAsO_2_ intoxication in mice.

### 3.6. GK Attenuated the Upregulation of mRNA Expression of MAPK (ERK, JNK, and p38)

**Figure 6:**
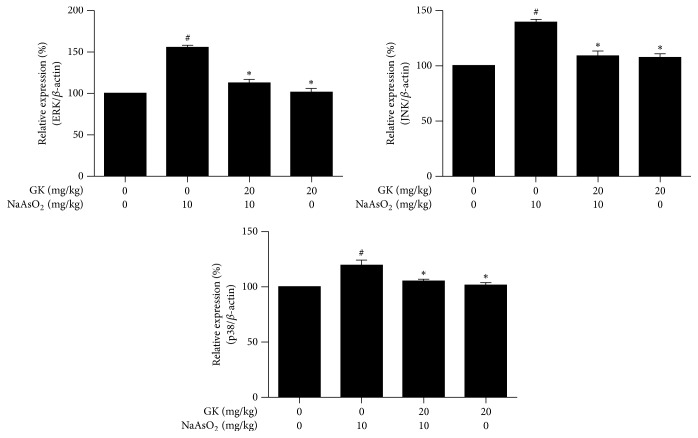
GK attenuated mRNA expression level of MAPK (ERK, JNK, and p38) in the liver tissue. In NaAsO_2_ (10 mg/kg) treated mice liver tissue, mRNA expression levels of ERK, JNK, and p38 were significantly upregulated while cotreatment with GK (20 mg/kg) markedly downregulated expression level. ^#^*p* < 0.05 when compared with the control and ^*∗*^*p* < 0.05 when compared with NaAsO_2_ treated. Data are expressed as mean ± SEM.

To determine whether MAPK signaling pathways were involved in NaAsO_2_-induced hepatotoxicity, we tested NaAsO_2_ stimulated expression and modulation of ERK, JNK, and p38 genes in liver tissue with and without GK cotreatment. We found treatment with NaAsO_2_ (10 mg/kg) markedly increased gene expression level of ERK, JNK, and p38 (155.34%, 139.16%, and 119.24%) compared to control (100%), and this elevated expression level was significantly attenuated (112.64%, 108.89%, and 104.94%, resp.) by cotreatment with GK (20 mg/kg) as compared with NaAsO_2_ alone. Thus, GK significantly inhibited mRNA expression of MAPK, which led to hepatoprotection in NaAsO_2_ intoxication in mice (Figure 6).

### 3.7. GK Suppressed MAPK (ERK1/2, JNK, and p38) and Caspase-3 Signaling Pathways and Maintained the Bax/Bcl-2 Expression Ratio

**Figure 7:**
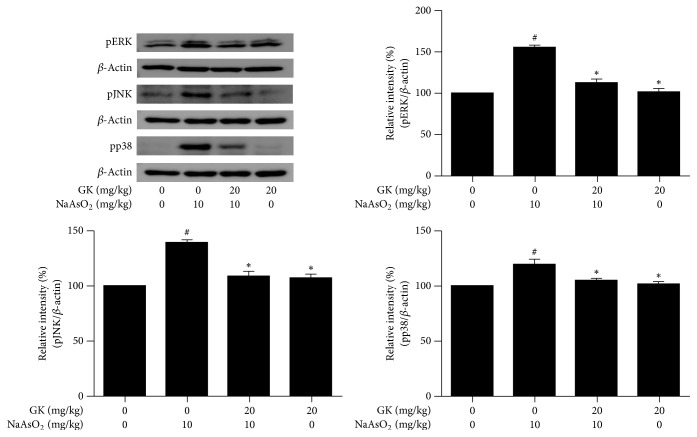
Immunoblotting analysis of MAPK (pERK, pJNK, and pp38) in liver tissue. Relative band intensity was measured as compared with *β*-actin. In NaAsO_2_ (10 mg/kg) treated mice, phosphorylation of ERK, JNK, and p38 was significantly upregulated while cotreatment with GK (20 mg/kg) markedly downregulated the expression level. ^#^*p* < 0.05 when compared with the control and ^*∗*^*p* < 0.05 when compared with NaAsO_2_ treated. Data are expressed as mean ± SEM.

**Figure 8:**
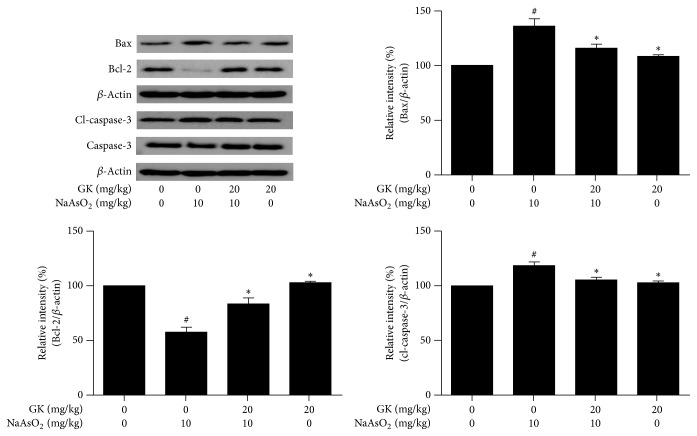
Immunoblot analysis of Bax, Bcl-2, and cleaved caspase-3 in liver tissue. Relative band intensity was measured as compared with *β*-actin. In NaAsO_2_ (10 mg/kg) treated mice, protein expression level of Bax and cleaved caspase-3 was significantly upregulated, and Bcl-2 was downregulated while cotreatment with GK (20 mg/kg) markedly downregulated expression level of Bax and cleaved caspase-3 and upregulated Bcl-2 expression. ^#^*p* < 0.05 when compared with the control and ^*∗*^*p* < 0.05 when compared with NaAsO2 treated. Data are expressed as mean ± SEM.

To understand the possible molecular pathways of hepatoprotection by GK extract, we evaluated MAPK family protein which played a crucial role in arsenic-induced hepatic damage and apoptosis. We analyzed the phosphorylation status of ERK1/2, JNK, and p38 and examined the effects of GK on the proapoptotic protein (Bax), the antiapoptotic protein (Bcl-2), and the triggering of a cleaved caspase-3 apoptotic factor by immunoblotting. We found treatment with NaAsO_2_ (10 mg/kg) significantly upregulated phosphorylation of the MAPK family protein expression compared to control. Phosphorylated (ERK1/2, JNK, and p38) protein level was remarkably attenuated by GK (20 mg/kg) cotreatment compared to NaAsO_2_ alone (Figure 7). Also, in comparison with the control, the expression level of Bax and Bcl-2 was increased and decreased markedly in the NaAsO_2_-treated group while being contrasted to the NaAsO_2_-treated group; the Bax/Bcl-2 expression was significantly decreased and increased respectively in the GK cotreatment group. Thus, the relative expression level of Bax/Bcl-2 family proteins was also significantly regulated by GK cotreatment (Figure 8). Since the apoptotic factor caspase-3 plays a vital role in hepatoprotection, we investigated cleaved caspase-3 protein level following treatment with NaAsO_2_. Expression of cleaved caspase-3 was markedly increased by treatment with NaAsO_2_. In contrast, cotreatment with GK (20 mg/kg) notably reduced the expression level of cleaved caspase-3 related to NaAsO_2_ alone (Figure 8). These results support that GK significantly inhibited phosphorylation of MAPK, well-regulated the Bax/Bcl-2 expression ratio, and concurrently suppressed the activation of apoptotic factor cleaved caspase-3 expression that led to the hepatoprotection.

## 4. Discussion

Sodium arsenite is a ubiquitous metalloid has been recognized as significant risk factor for public health concern. Globally, about 200 million people are exposed to inorganic arsenic compounds through underground contaminated drinking water [31]. Extensive studies have been undertaken to increase understanding of the mechanisms of arsenic intoxication with the goal of designing treatment and assigning proper levels of hazard to exposure. Much progress has been made in the development of equipment capable of removing detectable levels of arsenic and other heavy metals from drinking water. However, it is equally imperative to understand the molecular mechanisms of arsenic toxicity. The key to understanding the pathogenesis of these diseases lies in the myriad of cellular processes that are changed or damaged by arsenic compounds. Treatment preventing the hepatic damage may lead to prospective therapeutic strategies against the hepatotoxicity and GK extract may provide a novel therapeutic candidate. In this study, we revealed that GK can be used as a novel indigenous phytomedicine due to its strong hepatoprotective effects against NaAsO_2_-mediated oxidative stress in vitro and in vivo.

Phenolic and flavonoid are the most important plant secondary metabolites and possessing strong antioxidant capacity [32, 33]. The antioxidant ability of them was mainly due to their redox properties, which allow them to act as reducing agents, oxygen scavengers, and transition metal ions chelator [34]. In our study, we found a considerable amount of phenolic and flavonoid content of GK extract that may be the major contributor for the antioxidant role against oxidative stress-induced hepatic damage. NaAsO_2_-induced cytotoxicity is the common method for the measurement of the potential hepatoprotective role of antioxidants [35, 36]. We found cotreatment with the GK increased cell viability and improved the cell morphology. Cellular proliferation, DNA repair, methylation, interactions with thiol and phosphate groups, and generation of ROS and signaling pathways are all influenced by arsenic [37]. Upregulation of ROS generation by inorganic arsenic compounds is known to damage cell membranes via lipid peroxidation, which is a fundamental cellular deteriorating vector in liver cells [38]. Several studies have indicated that ROS production and elevated lipid peroxidation are combined effects of oxidative stress on the development of arsenic hepatotoxicity [39, 40]. In this study, NaAsO_2_ exposure to HepG2 cells resulted in a significant increase in both ROS and LDH level, which confirmed the oxidative damage. Cotreatment of GK markedly attenuated the elevation of both ROS and lactate dehydrogenase release in HepG2 cells. This might be due to the antioxidant properties of GK that defended against radical attacks and maintained the normal physiology of the cell membrane.

Because of its metabolic roles and association with the gastrointestinal tract, the liver is vulnerable to toxicity induced by xenobiotics [41]. Histopathology and serum biochemistry serve as an initial indicator of physiological dysfunction and the incidence of liver intoxication [42, 43]. To observe NaAsO_2_-induced liver alterations, we performed histopathology on liver tissues. We detected degeneration of the nucleus, enlargement of the portal vein, and blurring of the cytoplasm in NaAsO_2_-treated liver tissue when compared with untreated control. Improved hepatocyte architecture was found in GK cotreated mice as related to NaAsO_2_ alone which has been revealed by an earlier study [44]. We, therefore, tested the level of biochemical parameters (ALT and AST) to further support our results. Serum ALT and AST levels were both found to be significantly increased only in the NaAsO_2_ exposure group as compared with the control group. However, coadministration of GK along with NaAsO_2_ remarkably altered the serum biochemical parameters to near normal level. These data suggest that GK has the potential to reduce NaAsO_2_-induced hepatotoxicity. Moreover, cotreatment with GK significantly increased the body weight than NaAsO_2_ alone.

MAPK include a family of serine/threonine phosphorylating proteins that mediate a variety of signal transduction pathways and are involved in gene expression related to regulation of inflammation, cell proliferation, and cell death [45]. ERK, JNK, and p38 were activated in response to ROS production and mitochondrial dysfunction, which are usually related to cell apoptosis [46]. To consider the basic mechanisms of apoptosis in NaAsO_2_ intoxicated liver and the protective role of GK in this hepatic pathophysiology, we examined the expression level of ERK, JNK, and p38 by qPCR and immunoblot analyses. We found a marked increase in both mRNA and phosphorylated protein expression of MAPK by NaAsO_2_ intoxication. Our results demonstrated the ability of NaAsO_2_ to induce the phosphorylation of ERK1/2, JNK, and p38 in liver tissues which are in agreement with a previous study [13] and the enhancement of phosphorylation of MAPK plays an important role in the signaling pathway of apoptosis. We found that cotreatment with GK significantly downregulated the activation of ERK, JNK, and p38 indicating that this MAPK pathway was involved with the protection of liver damage by GK.

Oxidative stress-induced apoptosis is primarily executed by upregulation of proapoptotic (Bax) and downregulation of antiapoptotic (Bcl-2 family) proteins and release of cytochrome *c*, which initiates caspase-dependent cell death. The Bax/Bcl-2 ratio is correlated with cell apoptosis [47]. Bcl-2 functions to inhibit cell death, while Bax, which forms heterodimers with Bcl-2, acts to accelerate cell death [48]. Our results showed that Bax expression significantly increased while Bcl-2 expression significantly decreased in NaAsO_2_-induced hepatotoxicity. In contrast, cotreatment with GK inhibited the proapoptotic Bax and increased the antiapoptotic Bcl-2 expression, indicating diminished hepatic damage. Arsenic-induced mitochondrial damage by increased oxidative stress and reciprocal regulation of Bax/Bcl-2 association with activation of caspase-3 ultimately led to apoptotic cell death [49]. During apoptosis, proapoptotic Bcl-2 protein relocates to the surface of mitochondria and thereby permeabilization of the mitochondrial membrane with release of cytochrome *c*. Cytochrome *c* promotes the ATP-dependent formation of apoptosome by aggregation of caspase-9 activation. Caspase-9 then prompts the caspase cascade by stimulating caspase-3 leading to apoptosis [50]. We found that NaAsO_2_ exposure led to cleaved caspase-3 activation suggesting induction of apoptosis. However, the transcription level of apoptotic protein activated caspase-3 was significantly restored near normal condition by GK cotreatment. Therefore, our results revealed that GK considerably regulates NaAsO_2_-induced mitochondrial-dependent oxidative damage in the liver. A further experiment is needed to identify the active compounds in GK methanolic extracts involved in hepatoprotective activities.

## 5. Conclusions

Taken together, our results demonstrate that GK extract attenuated the NaAsO_2_-induced hepatotoxicity both in vivo and in vitro. GK appears to exert its hepatoprotective role by attenuating cytotoxicity and free radical generation in HepG2 cells. Moreover, GK restores the hepatic physiology and reduction in the mitochondrial-dependent hepatotoxicity. Thus, GK, a natural phytomedicine, may be a promising indigenous therapeutic agent for treatment of hepatic disorders by targeting oxidative stress.

## Figures and Tables

**Table 1:** Nucleotide sequence of the primers for qPCR.

Gene	Primers sequence (5′-3′)	Size (bp)	GenBank accession number
ERK	TCAGAGGCAGGTGGATCTCT	109	NM_011949.3
	ACGGGGAGGACTCTGTTTTT		
JNK	CGGAACACCTTGTCCTGAAT	93	NM_016700.4
	CACATCGGGGAACAGTTTCT		
p38	AGCCAATTCCAGTGTTGGAC	120	NM_011951.3
	TTCTGGGCTCCAAATGATTC		
*β*-Actin	AGAAGATCTGGCACCACACC	195	NM_007393.5
	TACGACCAGAGGCATACAGG		

**Table 2:** Total phenolic, flavonoid, and extraction yield of GK.

Plant extract	Total phenolics (mg GAE/g extract)	Total flavonoids (mg RU/g extract)	Total yield (%)
Methanolic extract	86.858 ± 0.132	182.964 ± 5.428	26.9

## Abbreviations

GK: *Geranium koreanum*
MTT: 3-(4,5-Dimethylthiazol-2-yl)-2,5-diphenyltetrazolium bromide
DMEM: Dulbecco's modified Eagle's medium
FBS: Fetal bovine serum
ROS: Reactive oxygen species
LDH: Lactate dehydrogenase
DMSO: Dimethyl sulfoxide
O.D: Optical density
qPCR: Quantitative real-time polymerase chain reaction
MAPK: Mitogen-activated protein kinases
ERK: Extracellular signal-regulated kinases
JNK: c-Jun N-terminal kinase
ALT: Alanine aminotransferase
AST: Aspartate aminotransferase
NBF: Neutral buffered formalin
SDS-PAGE: Sodium dodecyl sulfate-polyacrylamide gel electrophoresis
TBST: Tris-buffered saline
PBST: Phosphate-buffered saline
ECL: Enhanced chemiluminescence
T-PER: Tissue protein extraction reagent
PMSF: Phenylmethylsulfonyl fluoride
Na3VO4: Sodium orthovanadate.
